# CAML mediates survival of Myc-induced lymphoma cells independent of tail-anchored protein insertion

**DOI:** 10.1038/cddiscovery.2016.98

**Published:** 2017-05-29

**Authors:** Jennifer C Shing, Lonn D Lindquist, Nica Borgese, Richard J Bram

**Affiliations:** 1 Department of Pediatric and Adolescent Medicine, Mayo Clinic College of Medicine, Rochester, MN, USA; 2 Consiglio Nazionale delle Ricerche Institute of Neuroscience, Milan, Italy; 3 Department of Immunology, Mayo Clinic College of Medicine, Rochester, MN, USA

## Abstract

Calcium-modulating cyclophilin ligand (CAML) is an endoplasmic reticulum (ER) protein that functions, along with WRB and TRC40, to mediate tail-anchored (TA) protein insertion into the ER membrane. Physiologic roles for CAML include endocytic trafficking, intracellular calcium signaling, and the survival and proliferation of specialized immune cells, recently attributed to its requirement for TA protein insertion. To identify a possible role for CAML in cancer cells, we generated *Eμ-Myc* transgenic mice that carry a tamoxifen-inducible deletion allele of *Caml*. In multiple B-cell lymphoma cell lines derived from these mice, homozygous loss of *Caml* activated apoptosis. Cell death was blocked by Bcl-2/Bcl-x_L_ overexpression; however, rescue from apoptosis was insufficient to restore proliferation. Tumors established from an *Eμ-Myc* lymphoma cell line completely regressed after tamoxifen administration, suggesting that CAML is also required for these cancer cells to survive and grow *in vivo*. Cell cycle analyses of *Caml*-deleted lymphoma cells revealed an arrest in G2/M, accompanied by low expression of the mitotic marker, phospho-histone H3 (Ser10). Surprisingly, lymphoma cell viability did not depend on the domain of CAML required for its interaction with TRC40. Furthermore, a small protein fragment consisting of the C-terminal 111 amino acid residues of CAML, encompassing the WRB-binding domain, was sufficient to rescue growth and survival of *Caml*-deleted lymphoma cells. Critically, this minimal region of CAML did not restore TA protein insertion in knockout cells. Taken together, these data reveal an essential role for CAML in supporting survival and mitotic progression in Myc-driven lymphomas that is independent of its TA protein insertion function.

## Introduction

Calcium-modulating cyclophilin ligand (CAML) is an endoplasmic reticulum (ER) membrane protein that was originally identified based on its interaction with cyclophilin B.^1^ It is highly conserved in vertebrates and ubiquitously expressed in mammalian tissues. Although embryonic stem cells from *Caml*^−/−^ embryos proliferate normally, homozygous loss of *Caml* caused embryonic lethality in mice.^2^ Therefore, CAML is thought to play an important role in cell physiology. Previous studies have implicated CAML in multiple cellular processes, including intracellular calcium signaling,^1,3,4^ membrane trafficking,^2,5^ signal transduction,^6^ and suppression of aneuploidy via mitotic spindle function;^7^ however, the underlying mechanism of these observations is unknown.

CAML was recently implicated as a mediator of tail-anchored (TA) protein insertion in mammalian cells.^8^ TA proteins, estimated to represent ~3–5% of all integral membrane proteins,^9^ possess a single hydrophobic transmembrane domain (TMD) near the C terminus, which serves as a membrane anchor for proteins to localize to the appropriate organelle.^10,11^ Because these membrane anchors reside in close proximity to the C terminus, steric hindrance from ribosomes prevents access of chaperones to these hydrophobic residues before completion of translation. Thus, TA proteins are completely synthesized and handed off to chaperone machinery before being targeted to the ER membrane for insertion. These events have been characterized in recent years, with yeast studies having elucidated the GET pathway of TA insertion and mammalian studies continuing to focus on the homologous TMD recognition complex of 40 kDa (TRC40) pathway.

CAML was identified as an interactor of TRC40 from an unbiased screen, which led to the discovery that CAML is a component of the TRC40 membrane receptor.^8^ By interacting with TRC40, it was demonstrated that CAML and tryptophan-rich basic protein (WRB) bind and cooperate to receive TA proteins from TRC40 and to insert the substrates into the ER membrane. Since many TA proteins have crucial functions throughout the cell,^10^ this discovery suggested that CAML may regulate a multitude of cellular processes by mediating TA protein biogenesis.

Although CAML has been studied in the context of normal cell biology, the role of this protein in cancer is currently unknown. In the present study, we investigated CAML function by employing a mouse model of B-cell lymphoma with a tamoxifen-inducible, *Caml* deletion system. *Eμ-Myc* lymphoma cell lines established from these mice displayed robust activation of apoptosis and growth arrest due to *Caml* loss. Rescuing apoptosis was insufficient to restore proliferation of CAML-deficient cells, demonstrating dual roles for CAML in cell viability and growth. Impaired proliferation was unrelated to cell death and was caused by a cell cycle defect, because *Caml*-deleted lymphoma cells displayed an accumulation in G2/M phase. Unexpectedly, structure–function analyses defined the CAML N terminus as dispensable and the C terminus as sufficient for cell viability. Importantly, the CAML C terminus does not have TA protein insertion ability. Therefore, the survival and growth functions of CAML are independent of TA protein insertion, and implicate a novel role for the C-terminal, lipid bilayer resident domain of CAML.

## Results

### *Caml* deletion reduces proliferation of *Eμ-Myc* lymphoma cells

To investigate the function of CAML in c-Myc-driven B-cell lymphomas, we generated *Eμ-Myc* transgenic mice carrying floxed alleles of *Caml* and the *Cre-ER*^*T2*^ transgene, thus allowing tamoxifen-inducible deletion of *Caml*.^12,13^ Spleens collected from mice exhibiting signs of lymphoma (for example, tumor masses and hunched back) were used to establish cell lines. Without exception, these lines expressed typical B lymphocyte cell surface markers, verifying their nature as c-Myc-induced B lineage neoplasms (data not shown).

**Figure 1:**
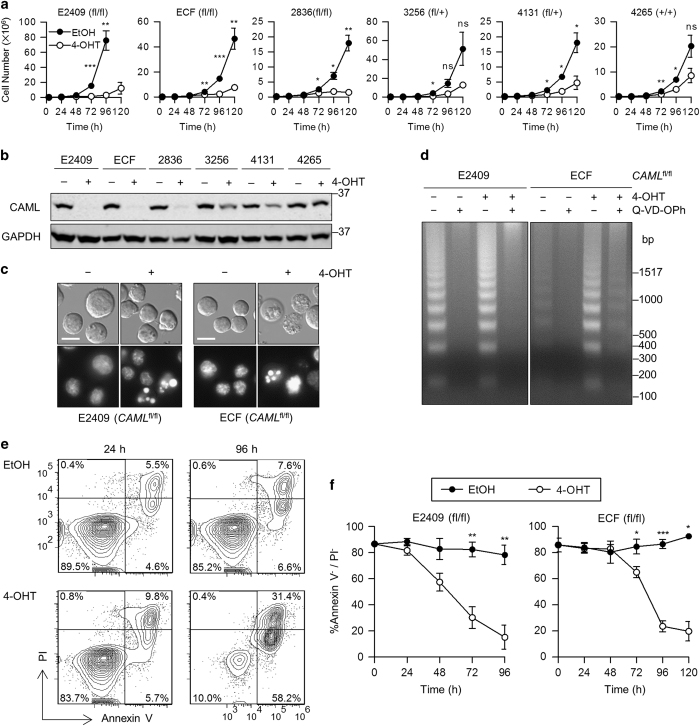
*Caml*-deleted *Eμ-Myc* lymphoma cell lines display impaired proliferation and apoptotic hallmarks. (**a**) Growth curves for *Eμ-Myc*;*Cre-ER*;*Caml*^fl/fl^, *Caml*^fl/+^, or *Caml*^+/+^ lymphoma cell lines treated with vehicle (EtOH) or 4-OHT. Live cells were counted at 24 h intervals by flow cytometry. (**b**) Loss of CAML protein in 4-OHT-treated *Eμ-Myc*;*Cre-ER*;*Caml*-floxed lymphoma cell lines. Lysates were collected from parallel cultures of **a** at 48 h, and CAML expression was analyzed by western blot. (**c**) Apoptotic nuclear condensation observed by Hoechst 33342 staining of 4-OHT-treated E2409 and ECF cells. Scale bar=10 *μ*m (**d**) DNA laddering in apoptotic 4-OHT-treated cells, with rescue by pan-caspase inhibitor treatment (Q-VD-OPh, 5 *μ*M). (**e**) Staining with annexin V and propidium iodide (PI) indicate apoptotic cell death in *Caml*-deleted cells. *Eμ-Myc*;*Cre-ER*;*Caml*^fl/fl^ (E2409) cell line was treated with vehicle (EtOH) or 4-OHT, and samples were stained at 24 and 96 h with annexin V-Cy5 and PI. (**f**) Live cells (annexin V-negative and PI-negative) declined over time following 4-OHT treatment of *Eμ-Myc*;*Cre-ER*;*Caml*^fl/fl^ cell lines compared with vehicle control. Values are mean±S.E.M. of three independent replicates. Statistical differences between EtOH and 4-OHT treatments, for each time point starting at 72 h, are denoted by ‘ns’ for not significant, *P<0.05, **P<0.01, and ***P<0.001.

Following induced deletion of *Caml, Eμ-Myc*;*Cre-ER*;*Caml*^fl/fl^ lymphomas exhibited a dramatic decline in viable cell proliferation (Figure 1a). The defect in proliferation was greatest in cells completely deleted for *Caml* (E2409, ECF, and 2836, referred to as ‘*Eμ-Myc Caml*^Δ/Δ^’), but was also present to a lesser degree in cells with *Caml* haploinsufficiency (3256, 4131, ‘*Eμ-Myc Caml*^Δ/+^’). Knockout specificity was verified by PCR (data not shown), and exhibited levels of CAML protein concordant with genotype (Figure 1b). We conclude that CAML is essential for proliferation of these Myc-dependent lymphoma cells.

### Apoptosis in *Caml*-deleted *Eμ-Myc* lymphoma cells

Previous studies demonstrated increased apoptosis in CAML-deficient lymphocytes;^13,14, 15^ thus, we hypothesized that apoptotic death was also occurring in these *Eμ-Myc*;*Cre-ER*;*Caml*^Δ/Δ^ lymphomas. To explore this possibility, we performed a series of assays to detect apoptotic hallmarks in *Caml*-deleted *Eμ-Myc* cells. Apoptotic bodies consisting of condensed chromatin were observed by Hoechst staining in 4-OHT-generated *Caml*^Δ/Δ^ cells (Figure 1c). In *Caml*^Δ/Δ^ ECF cells, classic apoptotic DNA fragmentation was detected, which was rescued by the presence of the broad spectrum caspase inhibitor Q-VD-OPh (Figure 1d). Since vehicle-treated ECF cells exhibited minimal DNA fragmentation, this method of apoptotic detection appeared to be specific for *Caml*-deleted ECF cells. 4-OHT-treated *Caml*^fl/fl^ E2409 cells showed DNA fragmentation increased above the basal amount seen in untreated cells due to spontaneous apoptosis.

Annexin V and PI staining provided further evidence for apoptosis and analysis of its kinetics, specifically due to 4-OHT treatment. Both *Caml*^Δ/Δ^ lymphoma lines, E2409 and ECF, displayed elevated annexin V-positive, PI-positive staining (Figure 1e, and data not shown). The kinetic profile of cell death showed that the percentage of live cells (annexin V-negative, PI-negative) declined faster in E2409 *Caml*^Δ/Δ^ cells than in ECF *Caml*^Δ/Δ^ cells (Figure 1f), with E2409 initiating cell death at ~36 h compared to ECF cells, which began dying at ~60 h following *Caml* gene deletion. Taken together, multiple lines of evidence demonstrated that CAML-deficient *Eμ-Myc* lymphoma cell lines undergo apoptotic cell death.

### CAML regulates viability and proliferation via distinct mechanisms

**Figure 2:**
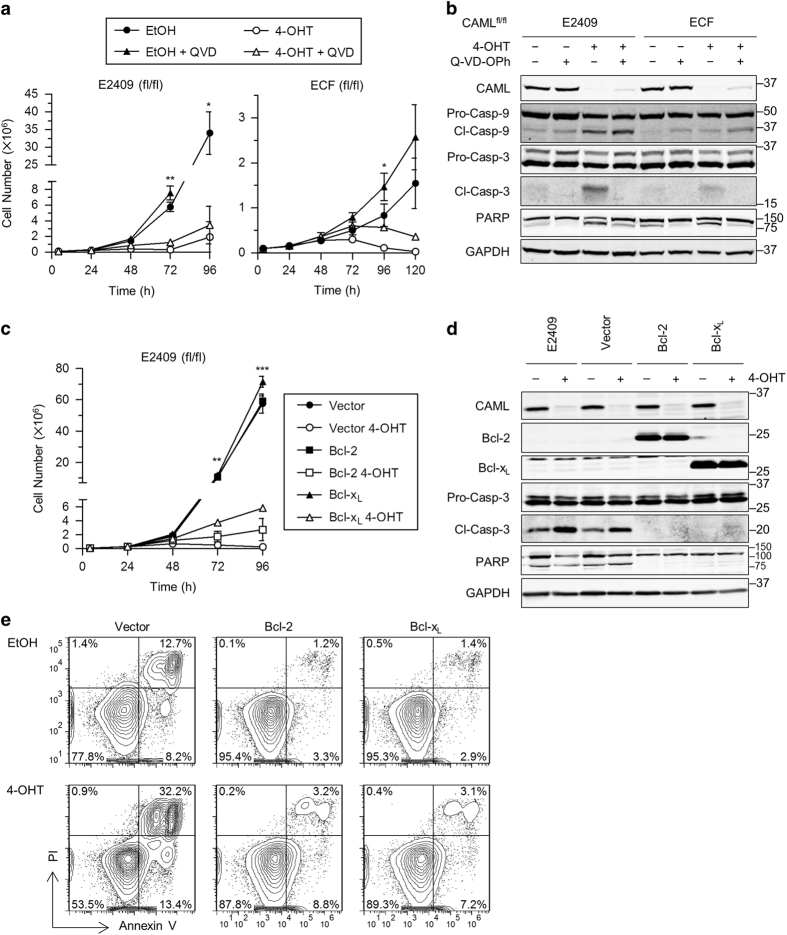
Inhibition of apoptosis in *Caml*-deleted cells does not restore proliferation. (**a**) Growth curves for *Eμ-Myc*;*Cre-ER*;*Caml*^fl/fl^ cell lines treated with vehicle (EtOH) or 4-OHT, and vehicle (DMSO) or caspase inhibitor Q-VD-OPh (QVD) (E2409, 25 *μ*M; ECF, 50 *μ*M). Values are mean±S.E.M. of two independent replicates from representative data that gave similar results. (**b**) Loss of caspase-3 and PARP cleavage by QVD treatment. Lysates were collected from parallel cultures of **a** at 48 h, and protein expression was analyzed by western blot. (**c**) Growth curves indicate proliferation is not rescued by Bcl-2/x_L_. Values are mean±S.E.M. of two independent replicates from representative data that gave similar results. (**d**) Western blot for parallel cultures of **c** showing loss of cleaved caspase-3 and PARP in Bcl-2/x_L_-overexpressing cells. (**e**) Annexin V and PI staining of *Caml*-targeted, Bcl-2/x_L_-overexpressing cells indicate rescue from cell death. *Eμ-Myc*;*Cre-ER*;*Caml*^fl/fl^ (E2409) Bcl-2/x_L_-overexpressing cells were treated with EtOH or 4-OHT and stained with annexin V and PI at 56 h. Statistical differences between EtOH and 4-OHT treatments, for time points starting at 72 h, are denoted by *P<0.05, **P<0.01, and ***P<0.001. Lack of significance is not indicated.

Pan-caspase inhibitor Q-VD-OPh was used to assess whether pharmacological caspase inhibition could rescue proliferation of *Eμ-Myc*;*Cre-ER*;*Caml*^Δ/Δ^ cells. For E2409 *Caml*^Δ/Δ^ cells, Q-VD-OPh did not reverse growth arrest, whereas ECF *Caml*^Δ/Δ^ cell numbers were partially rescued at 96 h post-4-OHT treatment (Figure 2a). Although western blot analysis showed that caspase-3 and PARP cleavage were suppressed by Q-VD-OPh treatment in *Caml*^Δ/Δ^ E2409 and ECF cells (Figure 2b), Q-VD-OPh did not inhibit caspase-9 cleavage. Thus, the mitochondrial pathway of apoptosis was active even in the presence of Q-VD-OPh.

We next asked if overexpression of the anti-apoptotic proteins Bcl-2 or Bcl-x_L_ might help restore proliferation. For this purpose, we used Bcl-2/x_L_ expressed in concert with green fluorescent protein (GFP) by the MigR1 retrovirus.^16^ Transduced E2409 *Caml*^fl/fl^ cells were sorted based on GFP expression to establish near-pure cultures of Bcl-2/x_L_ overexpressing cells (>95% GFP-positive). Interestingly, Bcl-2/x_L_ overexpressing *Caml*^Δ/Δ^ cells proliferated very poorly, although apoptosis and cell death were efficiently suppressed (Figure 2c). Similar to pharmacological caspase inhibition, western blotting of Bcl-2/x_L_ overexpressing *Caml*^Δ/Δ^ cells did not reveal caspase-3 or PARP cleavage (Figure 2d), and low percentages of annexin V-positive and PI-positive cells were observed (Figure 2e). Therefore, although apoptosis was effectively rescued, restoration of *Caml*^Δ/Δ^ cell proliferation was not achieved by caspase inhibition or anti-apoptotic protein overexpression. We conclude that CAML is required independently for both cell survival and for growth of *Eμ-Myc* lymphoma cells.

### *Eμ-Myc*-derived tumors require CAML for growth *in vivo*

Requirements for cell survival differ between culture conditions and in intact animals. To investigate this question, we adapted *Eμ-Myc*;*Cre-ER*;*Caml*^fl/fl^ E2409 cells to grow as solid tumors in mice. E2409 cells were subcutaneously injected into the hind leg of a C57BL/6 mouse, and the resulting tumor cells were collected, expanded *in vitro*, and cryopreserved for subsequent experiments. We verified that the adapted E2409 cells were indeed *Eμ-Myc* cells by cell surface staining and 4-OHT treatment, which completely depleted CAML protein (data not shown). Tumor allografts were established by subcutaneous injection of E2409 cells into the hind legs of athymic nude mice. When the tumors reached ~100 mm^3^, mice were randomized and injected intraperitoneally with vehicle or tamoxifen (50 mg/kg) to delete *Caml*.

**Figure 3:**
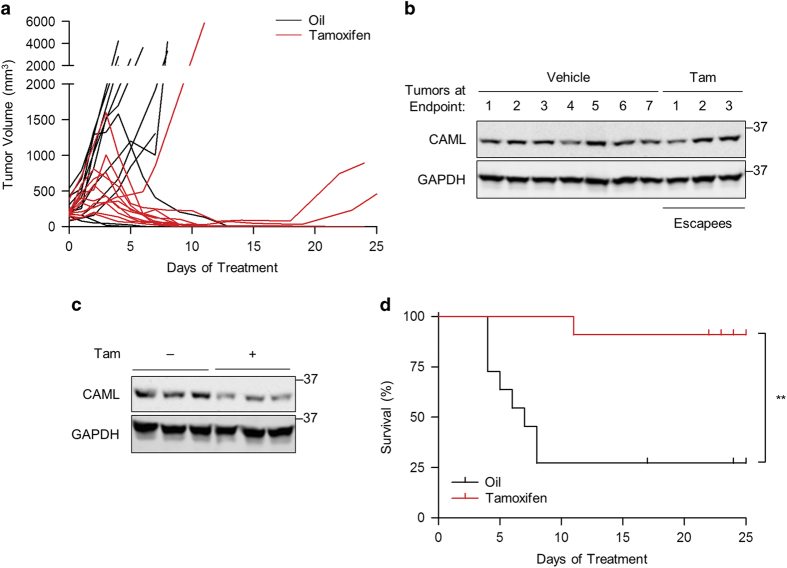
Genetic deletion of *Caml* causes lymphoma regression in athymic nude mice. Mice were injected with vehicle (corn oil) or tamoxifen (50 mg/kg), when lymphoma tumors derived from *Eμ-Myc*;*Cre-ER*;*Caml*^fl/fl^ cells (E2409) reached ~100 mm^3^. (**a**) Volumes of control (*n*=11) and tamoxifen-treated (*n*=11) tumors in nude mice. (**b**) Western blot for CAML in tumors at the time of killing for data in **a**. Each lane represents an individual mouse. (**c**) Western blot for CAML in tumors from mice treated with tamoxifen. (−) indicates untreated, (+) indicates mice treated with tamoxifen for 2 consecutive days and killed on the third day. Each lane represents an individual mouse. (**d**) Kaplan–Meier survival curves for vehicle and tamoxifen-treated groups in **a**. **P<0.01.

Tumors with tamoxifen-induced deletion of *Caml* exhibited rapid regression in the majority of mice (8 out of 11; Figure 3a). For the three tumors treated with tamoxifen that were unresponsive (one out of three) or that relapsed following regression (two out of three), all expressed levels of CAML protein at experimental end points comparable to the vehicle-treated controls (Figure 3b), presumably due to loss of *Cre* responsiveness to tamoxifen. Western blotting indicated CAML protein reduction in tumors due to tamoxifen treatment (Figure 3c), similar to that previously demonstrated in tissues examined for *Cre-ER*;*Caml*^fl/fl^ mice treated with tamoxifen.^14,17^ Kaplan–Meier survival curves plotted for tumor end points requiring killing showed a dramatic difference in survival with the vast majority of tamoxifen-treated mice alive at the conclusion of the experiment (*P*<0.01; Figure 3d). We conclude that CAML is essential for Myc-dependent lymphoma growth and survival *in vivo*.

### CAML loss causes G2/M arrest

**Figure 4:**
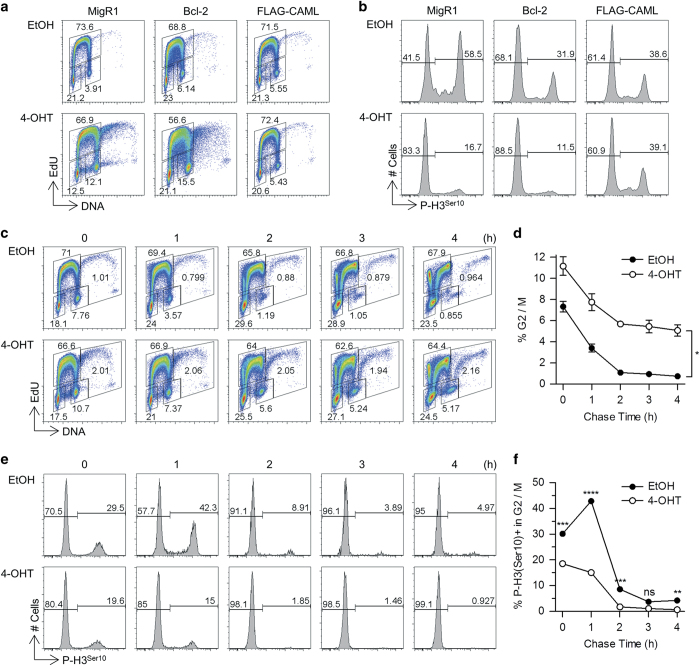
Accumulation of G2/M cells with decreased pH3 expression due to *Caml* deletion. Flow cytometric analysis of EdU incorporation, DNA content, and phosphorylated histone H3 on serine 10 (pH3) was performed for *Eμ-Myc*;*Cre-ER*;*Caml*^fl/fl^ (E2409) cells. For **a** and **b**, 1 h EdU incorporation was performed at 48 h after EtOH or 4-OHT treatment of empty vector (MigR1), Bcl-2 and FLAG-CAML-overexpressing cells. (**a**) Cell cycle profiles for transduced E2409 cells treated with EtOH or 4-OHT. (**b**) For G2/M gate in **a**, pH3 expression is displayed for transduced E2409 cells. For **c**–**f**, 30 min EdU incorporation was performed at 48 h after EtOH or 4-OHT treatment of E2409 cells expressing MigR1 empty vector. After EdU removal, samples were collected at indicated time points and later processed for EdU labeling, pH3 and DAPI staining. (**c**) Cell cycle profiles for pulse-chase MigR1 E2409 cells. (**d**) Kinetics of G2/M cells for pulse-chase in **c**. (**e**) pH3 expression for G2/M gate in **c**. (**f**) Kinetics of G2/M cells for pulse-chase in **e**. Statistical differences between EtOH and 4-OHT treatments are denoted by ‘ns’ for not significant, *P<0.05, **P<0.01, ***P<0.001, and ****P<0.0001.

Since blocking apoptosis did not restore growth of *Caml*-deleted *Eμ-Myc* cells, we postulated that CAML loss may impair cell cycle progression, in addition to inducing cell death. To test this hypothesis, we used S-phase 5-ethynyl-2′-deoxyuridine (EdU) incorporation and DAPI staining to evaluate cell cycle distribution in cells lacking *Caml*. Interestingly, we observed a ~three-fold increase in the percentage of cells in the G2/M phase at 24 h after activating deletion of the *Caml* gene (Figure 4a). This effect was not dependent upon selective death in G1 or S phases, because suppression of apoptosis by Bcl-2 did not substantially prevent the accumulation of G2/M phase cells upon loss of *Caml* (Figure 4a). Importantly, the upregulation of the G2/M subset was not related to tamoxifen or Cre activity, because *Cre-ER*;*Caml*^fl/fl^ cells transduced with a CAML expression vector did not exhibit G2/M population enrichment after drug-induced deletion of the endogenous floxed *Caml* genes (Figure 4a).

To further explore the nature of the block in cell cycle progression induced by CAML depletion, we stained cells for the mitotic chromosome condensation marker phospho-histone H3 on serine 10 (pH3).^18^ In total, 58.5% of control cells in G2/M were positive for pH3, whereas *Eμ-Myc Caml*^Δ/Δ^ cells had a dramatically lower proportion (16.7%) of pH3-positive cells (Figure 4b). Although pH3 may also be associated with apoptosis,^18,19^ we found that *Caml*-deleted cells that overexpressed Bcl-2 to block cell death also exhibited decreased pH3 (Figures 4b, 2d and e), indicating that loss of pH3-positive cells did not result from cell death. Loss of pH3 positivity in G2/M phase cells was not due to tamoxifen or Cre-mediated toxicity (Figure 4b, FLAG-CAML).

As CAML depletion caused an increase in cells falling within the G2/M gate but also reduced the relative percentage of mitotic cells, this buildup might represent either cells blocked prior to mitotic phase entry, or cells that failed to complete normal karyokinesis. To distinguish between these two possibilities, pulse-chase analysis was performed using a 30 min EdU incorporation interval, followed by 0, 1, 2, 3, or 4 h of culture in the absence of EdU. This allowed us to specifically monitor the disappearance of EdU-negative cells from the G2/M gate at 0 h without interference from an influx of new (EdU-positive) cells (that could be separately gated out).

*Caml*-deleted G2/M cells left the G2/M gate at a slower rate than control cells (Figure 4c). Control cells decreased by 10-fold (7.8–0.9%), whereas *Caml*-deleted cells decreased by two-fold (10.7–5.2%) over the 4 h chase period (Figures 4c and d). Furthermore, the proportion of pH3-positive cells in the G2/M gates decreased rapidly in both control and CAML-depleted cells over several hours, indicating that exit from mitosis was not delayed by loss of the gene (Figures 4e and f). There was no suggestion that entry of G2/M cells into the pH3-positive gate was significantly delayed in cells lacking CAML. Taken together, we conclude that *Caml* deletion in *Eμ-Myc* cells causes a defect in the cell cycle, most likely after cells exit from mitosis. However, as a significant number of mutant cells remained within the G2/M gate, we suspect that they were likely the product of failed cell division and had become tetraploid. Indeed, examination of the cell cycle plots revealed a significant increase in cells with a pattern consistent with a 4N-to-8N G1-to-S-to-G2/M distribution.

### Structure–function analysis of CAML deletion mutants

**Figure 5:**
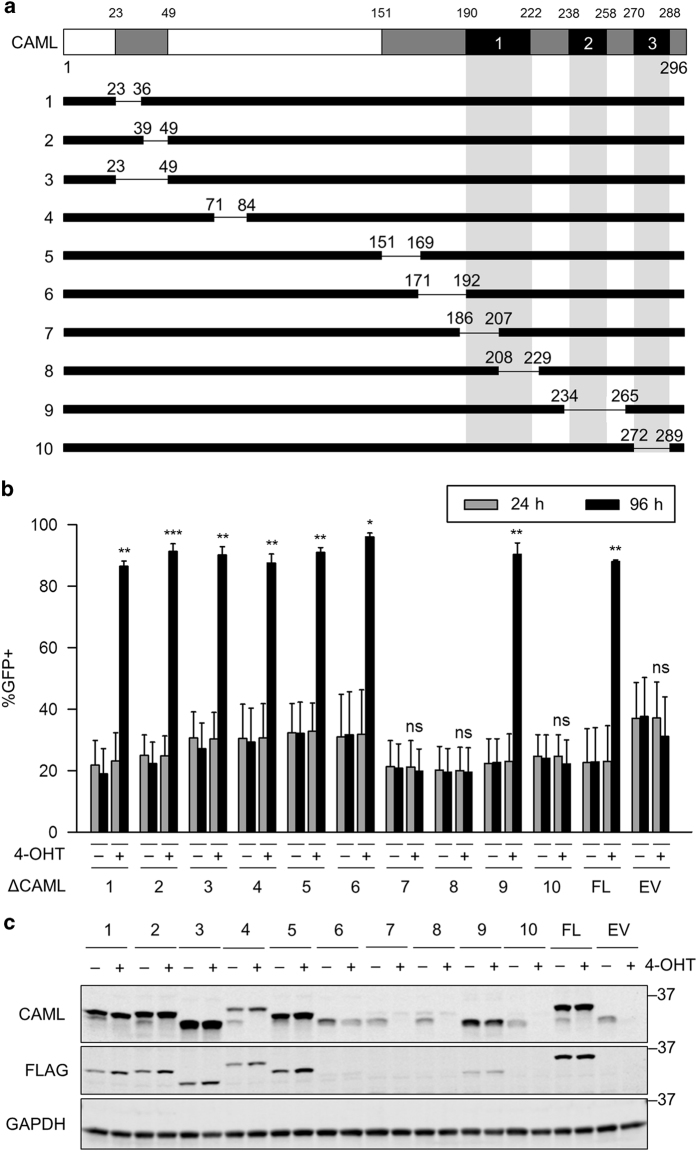
Rescue and expression analysis of *Caml*-deleted lymphoma cells. (**a**) Schematic representation of CAML deletion mutants (ΔCAML1–10). Mutants were subcloned into MigR1 vector with an N-terminal FLAG tag. Deleted amino acids are indicated. Gray=conserved regions, Black=transmembrane domains (TMD1–3). (**b**) ΔCAML1–6 and 9 rescue *Caml*-deleted lymphoma cells. *Eμ-Myc*;*Cre-ER*;*Caml*^fl/fl^ (E2409) cell line was transduced with the indicated CAML deletion mutants, treated with vehicle (−, ethanol) or 4-OHT (+), and measured for GFP expression at 24 and 96 h after treatment. FL=FLAG-CAML (full length), EV=MigR1 empty vector. Values are mean±S.E.M. of two independent replicates from representative data that gave similar results. (**c**) Western blot for CAML deletion mutants. Lysates were collected from parallel cultures of **a** at 48 h after 4-OHT treatment. Statistical differences between 24 and 96 h time points for 4-OHT-treated cells are denoted by ‘ns’ for not significant, *P<0.05, **P<0.01, and ***P<0.001.

To understand which CAML domains are needed for its roles in survival and growth, we performed structure–function analyses by deleting the endogenous *Caml* alleles and determining whether expression of CAML deletion mutants could rescue the cells from apoptosis. FLAG-tagged human CAML deletion mutants were cloned into the MigR1 retroviral vector, each lacking various stretches of ~10–30 amino acid residues (Figure 5a). MigR1 encodes GFP controlled by an internal ribosome entry site. Thus, we reasoned that expression of a deletion mutant with rescue ability would enable cells to outcompete untransduced GFP-negative *Caml*^Δ/Δ^ cells, and enhance the proportion of GFP-positive cells over time.

Surprisingly, all deletion mutants that removed portions of the cytoplasmic N terminus were able to rescue E2409 *Caml*^Δ/Δ^ cells (ΔCAML1–6) (Figure 5b). Moreover, ΔCAML9, lacking the second predicted TMD, rescued *Caml*-deleted cells. On the other hand, mutants with deletions that encompassed TMD1 (Δ7, 8) or TMD3 (Δ10) were unable to rescue *Caml*-deleted cells. Expression of protein from constructs ΔCAML7, 8, and 10 was much lower than those that restored survival, possibly reflecting instability rather than a functional defect (Figure 5c).

### CAML C terminus rescues *Caml*^Δ/Δ^ cells

**Figure 6:**
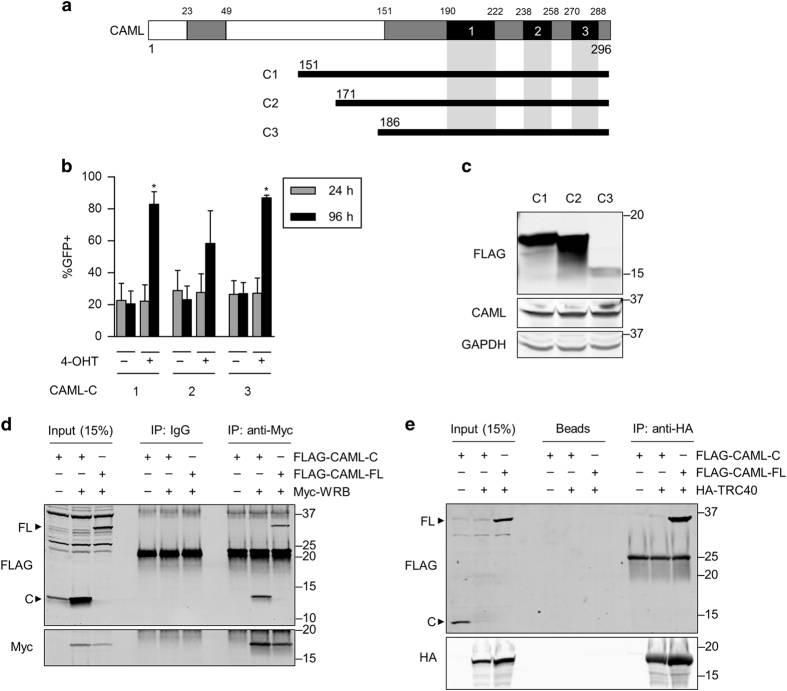
CAML C terminus is sufficient to rescue *Caml*-deleted cells, and binds to WRB but not TRC40. (**a**) Schematic representation of CAML C-terminal fragments. Truncation mutants were subcloned into MigR1 vector with an N-terminal FLAG tag. Expressed amino acids are indicated. Gray=conserved regions, Black=transmembrane domains (TMD1–3). (**b**) CAML-C1–C3 rescue *Caml*-deleted lymphoma cells. *Eμ-Myc*;*Cre-ER*;*Caml*^fl/fl^ (E2409) cell line was transduced with the CAML-C fragments, treated with vehicle (−, ethanol) or 4-OHT (+), and measured for GFP expression at 24 and 96 h after treatment. Values are mean±S.E.M. of two independent replicates from representative data that gave similar results. (**c**) Western blot for CAML-C fragments. HEK293T cells were transfected with CAML-C vectors and collected for lysate. (**d**) CAML-C binds to WRB. HEK293T cells were transfected with combinations of CAML-C3 (FLAG-CAML-C), full-length CAML (FLAG-CAML-FL), and Myc-WRB, collected for lysate, immunoprecipitated with anti-Myc antibodies, and analyzed by western blot. (**e**) CAML-C does not bind to TRC40. Same method as in **d** was performed except HA-TRC40 was transfected instead of Myc-WRB, and immunoprecipitation was performed using anti-HA-affinity matrix. Statistical differences between 24 and 96 h time points for 4-OHT-treated cells are denoted by *P<0.05. Lack of significance is not indicated.

Since CAML mutants lacking portions of the N terminus demonstrated rescue ability of *Caml*^Δ/Δ^ cells, we tested additional mutants that expressed only the C-terminal predicted TMDs of CAML (Figure 6a). Three truncations of CAML that encoded residues 151–296, 171–296, and 186–296 rescued *Eμ-Myc Caml*^Δ/Δ^ cells (Figure 6b), indicating that the cytosolic N terminus is dispensable for cell viability. Although these C-terminal mutants were not detected by western blot of E2409 cells (data not shown), we observed protein expression in HEK293T cells transfected with these truncations (Figure 6c). We conclude that a minimal region of CAML (186–296 hereafter ‘CAML-C’) is sufficient to support the survival of *Caml*^Δ/Δ^ cells.

Given that the CAML C terminus is sufficient for cell viability, we tested whether this region facilitates TA insertion as well. Initially, we performed co-immunoprecipitations with CAML-C to assess whether it is able to bind to WRB or TRC40. HEK293T cells were co-transfected with Myc-tagged WRB and CAML-C, or HA-tagged TRC40 and CAML-C and protein complexes were precipitated using anti-Myc or anti-HA antibodies. There was a clear interaction between Myc-WRB and CAML-C (Figure 6d); however, no significant binding occurred between HA-TRC40 and CAML-C (Figure 6e), in agreement with the results of Yamamoto and Sakisaka.^8^ Collectively, these data suggest that interaction between TRC40 and CAML is not required for viability, and raise the possibility that WRB and CAML binding may be essential for survival.

### TA protein insertion is not required for cell viability and growth

Because the CAML C terminus was sufficient to rescue *Caml*-deleted *Eμ-Myc* cells, we asked whether rescue correlates with ability of the C terminus to support TA protein insertion. We performed an *in vitro* translocation assay using permeabilized *Eμ-Myc*;*Cre-ER*;*Caml*^fl/fl^ (E2409) cells incubated with *in vitro*-synthesized protein substrate.^20^ The TMD of synaptobrevin requires TRC40 for insertion,^21^ whereas the control protein, cytochrome b5, spontaneously inserts into the ER membrane even in the absence of TA import machinery.^22^

**Figure 7:**
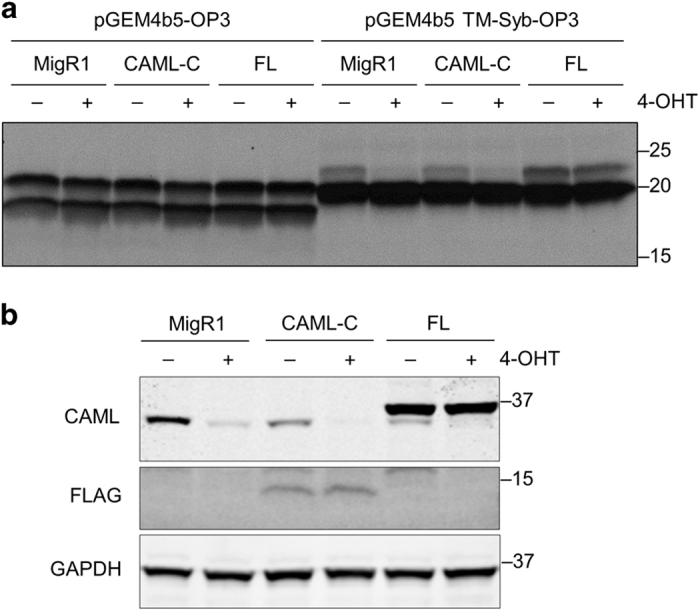
CAML C terminus does not restore TRC40-dependent TA protein insertion into the membrane. (**a**) *Eμ-Myc*;*Cre-ER*;*Caml*^fl/fl^ (E2409) cell line was transduced with empty vector (MigR1), CAML-C3 (CAML-C), or full-length CAML (FL), treated with vehicle (−, ethanol) or 4-OHT (+), and collected for permeabilization at 24 h after treatment. Plasmids encoding opsin-tagged cytochrome b5 (pGEM4b5-OP3) and the TMD of synaptobrevin-2 (pGEM4b5 TM-Syb-OP3) were subjected to *in vitro* transcription/translation. Reactions were incubated with semi-permeabilized, transduced E2409 cells, separated by SDS-PAGE, and visualized by autoradiography. (**b**) Western blot for parallel cultures in **a**.

As predicted, E2409 *Caml*^Δ/Δ^ cells did not mediate efficient, post-translational insertion of synaptobrevin (Figure 7a); whereas MigR1-mediated expression of full-length CAML, but not CAML-C, restored TA protein insertion activity (Figures 7a and b). In summary, these findings demonstrate that lymphoma cell survival requires CAML-C, but does not appear to depend upon functioning TA protein insertion machinery.

## Discussion

Although previous studies discovered that CAML is essential for growth of activated immune cells,^13,14, 15^ its function in mammalian TA insertion was only revealed more recently.^8^ For this study, we aimed to reconcile these distinct roles of CAML in cell viability and TA insertion using *Eμ-Myc* lymphoma cell lines with tamoxifen-inducible deletion of *Caml*. Our results indicate that CAML is required for the survival and growth of Myc-driven B-cell lymphomas, in culture and *in vivo*, likely by facilitating mitosis. Structure–function analyses suggest that the N-terminal domain of CAML, which interacts with TRC40, is dispensable for *Eμ-Myc* cell proliferation. Moreover, the C-terminal transmembrane region of CAML was sufficient for restoring survival of *Caml*^Δ/Δ^ cells. Although this region interacts with WRB, TA protein translocation was absent when the C terminus of CAML was exclusively expressed. Thus, our results strongly suggest that TA insertion is not responsible for the survival and growth functions of CAML in *Eμ-Myc* cells, and indicates that CAML possesses a TA insertion-independent function to support cell viability.

Our present study is the first to explore CAML function in a cancer model, and is consistent with previous findings in stimulated B and T lymphocytes, and thymocytes.^13,14,15,17^ Under conditional *Caml* deletion, these cell types showed increased apoptosis when they were activated by mitogens, similar to our results in *Eμ-Myc* lymphomas. Notably, CAML does not appear to function as a constitutive survival factor, because unstimulated cells (that is, thymocytes, mature B and T cells) did not activate apoptosis.^13,15,17^ Furthermore, embryonic stem cells prepared from *Caml*^−/−^ embryos were normal and proliferated rapidly.^2^ Although the *Eμ-Myc* cells in this study were not stimulated by exogenous factors, they exhibited rapid, basal growth, suggesting that proliferation of differentiated cells is necessary for detecting CAML-related phenotypes. The recent finding that rapamycin rescues CAML-deficient T lymphocytes from cytotoxicity by preventing cell division,^17^ aligns with our lymphoma model showing that *Caml*-deleted *Eμ-Myc* cells arrest in G2/M. Therefore, we reason that CAML facilitates cell cycle progression, which is apparent when cells are actively proliferating.

Our data using Bcl-2/x_L_ overexpression in *Caml*-deleted *Eμ-Myc* cells indicates that CAML regulates cell viability and proliferation via distinct mechanisms. Although Bcl-2 and Bcl-x_L_ possess C-terminal tails which could presumably impact the localization of these anti-apoptotic proteins following *Caml* deletion,^23^ the mechanism of Bcl-2/x_L_ translocation into the ER or mitochondrial membrane is unknown.^24^ Because downstream apoptotic events were inhibited by overexpression of Bcl-2/x_L_, even in the absence of CAML, it is reasonable to conclude that CAML has dual roles in viability and growth.

The *Eμ-Myc* model in our study used structure–function analyses to identify CAML domains responsible for cell survival and TA insertion. Though the cytoplasmic N-terminal domain of CAML interacts with TRC40 to receive TA proteins for ER translocation,^8^ we observed that the N terminus is dispensable for viability of *Eμ-Myc* cells. Furthermore, we discovered that the transmembrane C-terminal domain of CAML was sufficient for cell viability, and confirmed previous findings that the C terminus does not bind to TRC40.^8^ In the ER membrane, WRB and CAML form a receptor to receive TA proteins from TRC40 and facilitate their integration into the membrane.^8,25^ This characterization of a receptor complex agrees with our confirmation that the CAML C terminus interacts with WRB. Remarkably, the present results demonstrate that although the CAML C terminus possesses pro-survival function, it is unable to facilitate TA protein insertion. This means that proper insertion and localization of TA proteins is not how CAML is able to exhibit pro-survival properties. Although alternative TA insertion pathways, such as spontaneous or chaperone-mediated translocation, may compensate for the loss of TA insertion,^26,27,28,29^ the TRC40 pathway targets integration of TA proteins with more hydrophobic TMDs and is the most widely accepted method of TA insertion to date.^30^

Previously, CAML was reported to regulate the mitotic spindle assembly checkpoint.^7^ This study detected impaired proliferation in mouse embryonic fibroblasts with *Caml* deletion accomplished by retroviral introduction of *Cre* recombinase. Whereas apoptosis and senescence were excluded as potential causes of decreased growth, *Caml*^Δ/Δ^ mouse embryonic fibroblasts displayed a weaker spindle assembly checkpoint and severe mitotic spindle dysfunction leading to chromosome missegregation. Our current results in CAML-deficient *Eμ-Myc* cells point to defects in mitosis, with a higher proportion of cells in G2/M phase and decreased numbers of pH3-positive cells. These observations were similar in Bcl-2-overexpressing *Caml*-deleted cells, therefore excluding the possibility that apoptosis causes the G2/M arrest. Because the cells have 4N DNA and lack the pH3 marker of mitosis,^31^ we postulate that the depressed proportion of pH3-positive cells reflects a post-mitotic defect, such as failed cytokinesis.

Our conclusion that CAML functions in cell division in a TA-independent manner agrees with a large body of evidence pointing to numerous roles of CAML in physiology. These studies revealed that CAML mediates membrane trafficking,^2,5^ signal transduction,^6^ and calcium signaling.^1,3,4^ In support of the possibility of pleiotropic CAML functions are recent studies in rat liver microsomes and tissue culture cells, indicating that endogenous CAML levels are in ~five-fold molar excess over endogenous WRB.^32^ Interestingly, siRNA-mediated CAML knockdown reduced WRB levels by destabilizing WRB mRNA, and not by affecting WRB protein stability. These findings, combined with previous research and our current study, suggest that the extra CAML in cells can be explained by a TA protein insertion-independent function. We cannot exclude the possibility that WRB and CAML cooperate in a TA-independent capacity, because CAML-C and WRB interact but do not facilitate TA insertion. Thus, it remains to be determined whether CAML with WRB or in a WRB-free pool has pro-survival properties.

As a whole, we have revealed that CAML is critical for B-cell lymphoma survival and proliferation by promoting progression through G2/M phase in a manner independent of TA protein insertion. Future studies should seek to identify the precise role of CAML beyond TA protein insertion, and investigate the biochemical link between pro-survival functions and ER translocation.

## Materials and methods

### Mice

*Eμ-Myc* transgenic,^12^ *Cre* recombinase-estrogen receptor fusion targeted to the *Rosa26* locus (*Rosa26-Cre-ER*^*T2*^),^33^ and *Caml*^floxed^ mice have been described previously.^13^ *Eμ-Myc*;*Cre-ER*^*T2*^;*Caml*^fl/fl^ and *Caml*^fl/+^ mice were generated by crossing *Rosa26-Cre-ER*^*T2*^ to *Caml*^fl/fl^ mice, bearing loxP sites flanking exon 2 of *Caml*. The progeny were crossed to *Eμ-Myc* transgenic mice and bred on a C57BL/6 genetic background. Animal work was approved by the Mayo Clinic Institutional Animal Care and Use Committee.

### Establishment of *Eμ-Myc*;*Cre-ER*^*T2*^;*Caml*^floxed^ cell lines

Spleens were collected from *Eμ-Myc*;*Cre-ER*^*T2*^;*Caml*^fl/fl^ or *Caml*^fl/+^ mice bearing lymphomas, and total splenocytes were isolated by pressing spleens through mesh strainers (40 *μ*m). Collected cells were cultured in RPMI-1640 supplemented with 10% fetal bovine serum (Seradigm, Radnor, PA, USA), 100 units/ml penicillin, 100 *μ*g/ml streptomycin, 2 mM L-glutamine, and 50 *μ*M *β*-mercaptoethanol (Life Technologies, Waltham, MA, USA).

### Induction of *Caml* deletion

To induce *Caml* deletion, cells were counted and treated with vehicle (100% ethanol) or 250 nM 4-hydroxytamoxifen (Sigma-Aldrich, St. Louis, MO, USA) for 4 h at a density of 5×10^5^ cells/ml. Treated cultures were centrifuged and resuspended in fresh medium with E2409 cells at 0.7–1×10^5^ cells/ml and all other cell lines at 2×10^5^ cells/ml. At 24 h intervals, cell lines were counted and replenished with medium to achieve optimal densities for log phase growth.

### Cell culture

HEK293T cells (Invitrogen, Waltham, MA, USA) were cultured in high glucose DMEM (Life Technologies). PLAT-E cells (provided by Dr Kay Medina) were cultured in high glucose DMEM under antibiotic selection with 1 *μ*g/ml puromycin and 10 *μ*g/ml blasticidin S (Sigma-Aldrich). These culture media were supplemented with 10% fetal bovine serum (Seradigm), 100 units/ml penicillin, 100 *μ*g/ml streptomycin, 2 mM L-glutamine (Life Technologies), and incubated at 37 °C with 5% CO_2_.

### Cell counting and sorting

Live cell counting was conducted by collecting equal volumes of cells and gated based on forward and side scatter (Accuri C6, BD Biosciences, San Jose, CA, USA). Cell sorting was completed based on GFP expression (FACSAria, BD Biosciences), with appropriate recovery time for cultures before subsequent assays.

### Apoptotic DNA ladder

Total genomic DNA was isolated by DNeasy Blood & Tissue Kit (QIAGEN, Hilden, Germany) from frozen cell pellets. DNA samples were quantified by absorbance and subjected to agarose gel electrophoresis.

### Nuclear morphology

Cells were centrifuged, resuspended in medium containing 0.75 *μ*g/ml Hoechst 33342 (Sigma-Aldrich), and incubated for 15 min at 37 °C before microscopic analysis. Caspase inhibitor Q-VD-OPh was purchased from Sigma-Aldrich.

### Annexin V and PI staining

Cells were washed once with 1×PBS, washed once with 1×binding buffer (10 mM HEPES (pH 7.4), 140 mM NaCl, 2.5 mM CaCl_2_), and then stained with 1 : 50 annexin V-Cy5 (BD Biosciences) and 0.5 *μ*g/ml propidium iodide (Sigma-Aldrich) in 1×binding buffer.

### Plasmids and transfection

For CAML deletion mutants, CAML cDNA was amplified by PCR and deletions were created by sequential overlap extension PCR.^34^ Products were digested by appropriate restriction enzymes and subcloned into pMigR1 containing a FLAG tag, so proteins would express a FLAG tag fused to the N terminus. pReceiver expression vectors encoding N-terminal-tagged Myc-WRB (EX-I0576-M43) and 3xHA-TRC40 (EX-T2997-M06) were obtained from Genecopoeia (Rockville, MD, USA). Vector sequences were verified by Sanger sequencing.

### Retrovirus generation and infection

PLAT-E cells were transfected with 12 *μ*g retroviral vector using Lipofectamine 2000 (Life Technologies). Viral supernatants were collected 48–72 h post transfection and filtered (0.45 *μ*m). Cells were infected with virus by centrifugation for 90 min in medium containing 10 mM HEPES and incubated overnight. The medium was removed after incubation and transduced cells were analyzed for GFP expression.

### Western blotting

Cells were washed with cold 1×PBS, and lysed on ice for 20 min in modified radioimmunoprecipitation assay (Mod-RIPA) buffer consisting of 1% NP-40, 0.25% Na-deoxycholate, 50 mM Tris (pH 7.5), 150 mM NaCl, 5 mM EDTA, as well as protease and phosphatase inhibitor cocktails (Calbiochem, Billerica, MA, USA). Cellular debris was removed by centrifugation at 21 000×*g* for 10 min at 4 °C. Protein was quantified by Bradford assay (Bio-Rad, Hercules, CA, USA), separated by SDS-PAGE, and transferred to PVDF membranes (Millipore, Billerica, MA, USA). Membranes were blocked in Seablock (Millipore), incubated with primary and secondary antibodies, and visualized by infrared imaging (Odyssey, LI-COR Biosciences, Lincoln, NE, USA).

### Antibodies

Rabbit polyclonal antibodies to human CAML (1–189) have been described.^4^ Antibodies to Bcl-2 (492) and Bcl-x_L_ (634) were obtained from Santa Cruz Biotechnology (Dallas, TX, USA). Antibodies to caspase-3 (9665), caspase-9 (9508), PARP (9542), and FLAG (2368) were obtained from Cell Signaling Technology (Danvers, MA, USA). Anti-GAPDH (AB2302) was obtained from Millipore. Antibodies to c-Myc (06–549) and HA (05-902R) were from Upstate (Billerica, MA, USA). Rabbit polyclonal antibody to FLAG (F7425) and mouse monoclonal antibody to FLAG (F1804) were obtained from Sigma-Aldrich.

### Cell cycle analysis

The Click-iT EdU Alexa Fluor 647 Flow Cytometry Assay Kit was obtained from Molecular Probes (Waltham, MA, USA). Cells were treated with 10 *μ*M EdU for 1 h (single time point) or 30 min (pulse-chase). Incorporated cells were collected, washed, fixed with 1% paraformaldehyde, and permeabilized with saponin-based wash reagent. For phospho-histone H3 (Ser10) detection, cells were incubated with PE-pH3 antibody (5764, Cell Signaling Technology) for 1 h at room temperature.

### Tumor studies

A male C57BL/6 mouse was injected subcutaneously with E2409 lymphoma cells (*Eμ-Myc*;*Cre-ER*;*Caml*^fl/fl^) into the right hind leg. When a tumor was established, the cells were collected, cultured, and frozen. For the animal studies in nude mice, cells were thawed, expanded in culture, and 1×10^6^ cells were injected subcutaneously into the hind leg of male, athymic nude mice at 14–16 weeks old (Envigo, Indianapolis, IN, USA). When tumors reached ~100 mm^3^, mice were randomized into the vehicle (corn oil) or treatment group (*n*=11/group), and injected intraperitoneally with oil or tamoxifen (50 mg/kg) for 3 consecutive days, followed by a day of rest, and thereafter, cycling between 2 consecutive days interspersed with a rest day. When available at end points, whole tumors were collected for western blot. Tumors were measured by digital calipers, and volumes were calculated by (length×width×height)×(π/6).

### Co-immunoprecipitation

HEK293T cells were transfected using jetPRIME reagent (Polyplus Transfection, Illkirch, France), washed once in 1× PBS, and lysed in 0.1% Triton X-100, 20 mM HEPES-NaOH (pH 7.5), 150 mM NaCl, and 10% glycerol containing protease inhibitor cocktail (Calbiochem) at 24–36 h after transfection. After centrifugation at 21 000×*g* for 10 min at 4 °C, lysates were quantified by Bradford assay (Bio-Rad). For all immunoprecipitations, a total of 500 *μ*g of whole cell lysate was incubated with beads/resin.

For immunoprecipitation using anti-Myc antibody, 5 *μ*g of anti-Myc (M4439, Sigma) or non-specific IgG antibody (12–371, Millipore) was incubated with lysate for 2 h at 4 °C. Protein G magnetic beads (Dynabeads, Invitrogen) were added and incubated for 1 h at 4 °C. For immunoprecipitation using anti-HA-affinity matrix (Roche, Basel, Switzerland), 30 *μ*l of resuspended matrix (or agarose beads as a control) was incubated with lysate for 2 h at 4 °C. Following multiple washes, the immunoprecipitated proteins were denatured and eluted in Laemmli sample buffer (Bio-Rad), and analyzed by SDS-PAGE and western blotting.

### ER translocation

Plasmids (pGEM4 b5-OP3 and pGEM4 b5-TM-Syb-OP3) for transcription/translation were provided by Dr Nica Borgese and subcloned previously.^35^ The TnT SP6 Transcription/Translation System (Promega, Madison, WI, USA) containing rabbit reticulocyte lysate was used to synthesize insertion substrates in the presence of [^35^S]methionine (1000 Ci/mmol) (PerkinElmer, Waltham, MA, USA) for 90 min at 30 °C. Translation reactions were inhibited by cycloheximide (0.3 mg/ml) and centrifuged at 100 000×*g* to sediment ribosomes. Cells, permeabilized with 0.004% digitonin in KHM buffer (110 mM KOAC, 20 mM HEPES (pH 7.2), 2 mM Mg(OAC)_2_), were added to the translation reaction containing energy mix (dATP, dGTP, creatine kinase, and creatine phosphate). Translocation reactions were incubated for 1 h at 32 °C, lysed and denatured in Laemmli sample buffer (Bio-Rad), then subjected to SDS-PAGE and autoradiography.

### Statistical analysis

Line and bar graphs display mean±S.E.M. The log-rank test was performed for the Kaplan–Meier survival curves, and all other analyses were performed using the unpaired Student’s *t*-test (GraphPad Prism, La Jolla, CA, USA).
